# Early outcomes of robotic vs open living donor right hepatectomy in a US Center

**DOI:** 10.1007/s00464-024-11469-4

**Published:** 2025-01-08

**Authors:** Yuzuru Sambommatsu, Vinay Kumaran, Daisuke Imai, Kush Savsani, Aamir A. Khan, Amit Sharma, Muhammad Saeed, Adrian H. Cotterell, Marlon F. Levy, Seung Duk Lee, David A. Bruno

**Affiliations:** 1 Division of Transplant Surgery, Department of Surgery, Hume- Lee Transplant Center, Virginia Commonwealth University School of Medicine, Richmond, VA USA; 2 Department of Surgery, Virginia Commonwealth University School of Medicine, Richmond, VA USA

**Keywords:** Robotic surgery, Living donor liver transplant, Living donor hepatectomy

## Abstract

**Background:**

Robotic living donor hepatectomy offers potential advantages but has been limited to high-volume centers, primarily in Asia and the Middle East. We report our experience establishing a robotic living donor right hepatectomy program in a U.S. center with low LDLT volume and no prior laparoscopic donor hepatectomy experience and analyze early outcomes.

**Methods:**

This retrospective cohort study analyzed 37 living donor right hepatectomies (13 robotic [including one open conversion], 24 open) performed between June 2022 and February 2024.

**Results:**

The robotic group had longer operative times (median [range], 451 [374–568] minutes vs 368 [276–421] minutes; *P* < 0.001) but less blood loss (median [range], 200 [50–700] mL vs 900 [300–2500] mL; *P* < 0.001). One case required unplanned open conversion due to gas embolism. Two hematomas/bleeding (Clavien–Dindo grade IIIB) occurred in the robotic group, but no biliary complications. Comprehensive Complication Index, liver function tests, and hospital stays were similar between the two groups, with no 90-day graft failure/mortality.

**Conclusion:**

With extensive surgical experience in both open donor hepatectomy and robotic surgery, along with meticulous preparation as a team, U.S. centers with lower LDLT volume and no laparoscopic experience can safely implement robotic living donor right hepatectomy, achieving comparable short-term outcomes to the open approach. Further research on long-term outcomes and donor quality of life is necessary.

**Supplementary Information:**

The online version contains supplementary material available at 10.1007/s00464-024-11469-4.

The ethical dilemma of living donor liver transplantation (LDLT) lies in subjecting healthy individuals to surgery and its associated risks solely for organ donation. Much of the morbidity associated with open donor hepatectomy is related to its extensive surgical incisions and includes pain, surgical site infection, and hernia [1, 2]. Ensuring donors' safety and quality of life (QOL) is paramount in LDLT, prompting the evolution of the minimally invasive donor hepatectomy technique. In recent years, robotic surgical systems have gained attention. It offers several advantages over laparoscopic surgery, including better ergonomics, superior stability, magnified three-dimensional vision, and a shorter learning curve [3, 4].

In 2012, Giulianotti et al. [5] at the University of Illinois-Chicago performed the first robotic living donor right hepatectomy. Since then, several centers have published studies on the safety and efficacy of robotic living donor hepatectomy. However, these reports have primarily come from certain high-volume centers in Asia and the Middle East, with no large cohort studies reported from the USA [6–10]. Moreover, most of them had extensive experience in the laparoscopic approach before transitioning to the robotic approach. It remains unclear whether robotic living donor hepatectomy can be safely conducted while ensuring favorable outcomes in facilities that are not high-volume centers for LDLT and Hepato-pancreato-biliary (HPB) surgery and lack experience in the laparoscopic approach.

In January 2023, we launched a robotic living donor hepatectomy program after thorough preparation beginning in 2022. To date, we have successfully performed 12 fully robotic living donor right hepatectomies. This study aims to share our experience in establishing a robotic donor hepatectomy program and to analyze the early outcomes. To our knowledge, this is the first comprehensive report on robotic living donor hepatectomy from the USA.

## Materials and methods

### Preparation of the program

At our institution, we perform approximately 160 deceased donor liver transplantations, 25 LDLTs, and 40 HPB surgeries annually. We have been performing robotic liver resections since 2016, but donor hepatectomies have been performed through open surgery, with no prior experience in laparoscopic donor hepatectomy. To ensure maximum donor safety, open donor hepatectomies have been performed by two experienced transplant surgeons working together. When initiating our robotic living donor hepatectomy program, these same two surgeons continued their collaboration. The surgeon with 20 years of HPB and transplant experience and 5 years of robotic surgery experience including hepatectomies and living donor nephrectomies was selected as the console surgeon performing the procedure, while the senior surgeon with 700 LDLT experience sat at the second console, providing guidance, particularly in determining the dissection lines for vessels and bile ducts. The bedside surgeon role was assigned to a junior surgeon who had recently completed a transplant and HPB fellowship. The team also included a surgical assistant who was well versed in robotic surgery. Over the course of one year, the entire team prepared for the robotic donor hepatectomy program. Preparation involved accumulating experience in robotic liver resections for liver tumors and cysts, including our first fully robotic right hepatectomy for a giant hemangioma. Additionally, we participated in observational visits to high-volume centers in Korea for three weeks. Our first three cases were performed as hybrid procedures, where hilar dissection, liver mobilization, and half of the parenchymal transection were conducted robotically, followed by a planned open conversion. The 4th case was successfully completed with a fully robotic approach. The 5th case underwent an unplanned open conversion due to concerns of gas embolism. The 6th to 16th cases were successfully completed with a fully robotic approach, resulting in a total of 12 fully robotic cases being completed.

### Evaluation of the donor and selection criteria for the robotic approach

Our selection criteria for living donors include age < 60 years old, no major comorbidities, < 20% steatosis, expected donor remnant liver volume > 30%, and expected graft-to-recipient weight ratio (GRWR) > 0.7%. We consider the right liver graft as the first choice. All living donor candidates undergo computed tomography (CT) angiogram and magnetic resonance imaging (MRI)/magnetic resonance cholangiopancreatography (MRCP) to assess vascular anatomy, volumetry, fat fraction, and biliary anatomy. For the robotic approach, we initially set stringent criteria, including age < 40 years, BMI < 25, estimated graft volume < 800 mL, and standard biliary or vascular anatomy, to ensure donor safety. After successfully completing the first four fully robotic cases, we expanded the criteria to include donors with biliary and vascular anatomic variations (Supplementary Table 1).

### Surgical technique and intraoperative management

**Fig. 1:**
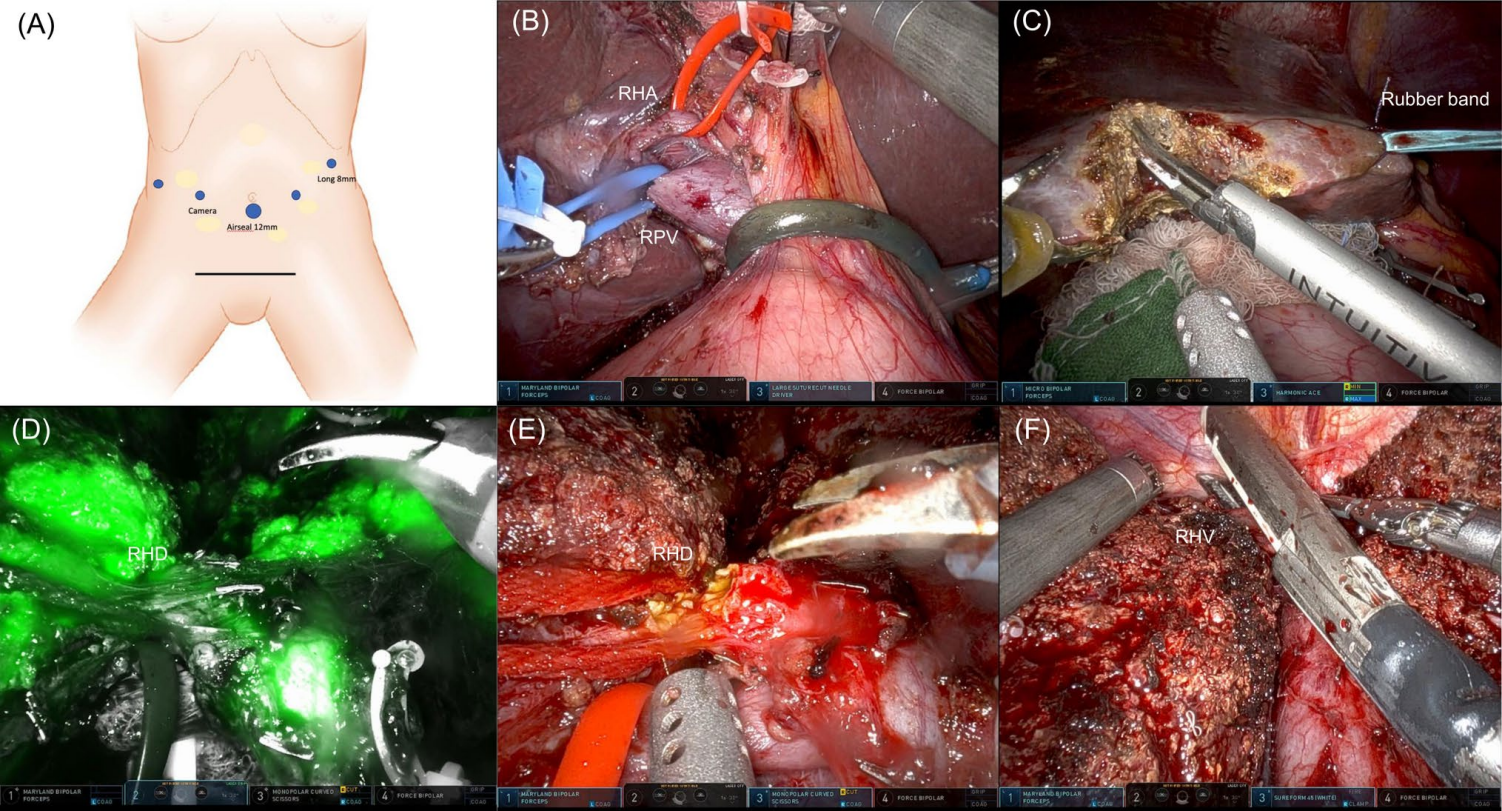
Surgical technique for robotic living donor right hepatectomy. **A** Port placement. **B** Identification of the right hepatic artery and portal vein. **C** Parenchymal transection using rubber band suspension method. **D** and **E** Bile duct division using ICG cholangiography. **F** Stapling of the right hepatic vein. *RHA* right hepatic artery, *RHD* right hepatic duct, *RHV* right hepatic vein, *RPV* right portal vein

Informed consent was obtained from each of the donors. Our group is currently preparing a separate manuscript that will detail the surgical techniques. In summary, robotic procedures were performed using the da Vinci Xi platform (Intuitive Surgical). Port placement is shown in Fig. 1A. First, hilar dissection was performed following cholecystectomy, and the right hepatic artery and right portal vein were isolated and looped with a vessel loop (Fig. 1B). Next, parenchymal transection was performed using the Harmonic scalpel and the Micro bipolar forceps, with the help of the rubber band suspension method [11] (Fig. 1C). After most of the parenchymal transection was completed, the right hepatic duct was identified and divided with the help of indocyanine green (ICG) cholangiography (Fig. 1D, E). The right hepatic duct stump was closed with a running 5–0 PDS suture. Once the parenchymal transection and isolation of the right hepatic vein were completed, a Pfannenstiel incision was made, and a specimen bag was introduced into the abdomen. After administering heparin, the right hepatic artery was ligated using a tie and clip, and the right portal vein, right hepatic vein, and inferior vena cava ligament were divided with a vascular stapler (Fig. 1F). The graft was retrieved through the Pfannenstiel incision.

In our center, intraoperative cell salvage is routinely used during open donor hepatectomy. The decision to return salvaged blood is made by anesthesiologists based on the amount of blood loss and the donor's hemodynamics. For robotic approach, considering the typically low blood loss, cell salvage is not routinely prepared.

### Study design

This cohort study was a retrospective analysis of all patients who underwent living donor hepatectomy at Virginia Commonwealth University Hume-Lee Transplant Center (Virginia, U.S.A.) between June 2022 and February 2024. This study was conducted in full compliance with the Declaration of Helsinki and was approved by the Institutional Review Board of Virginia Commonwealth University. This study followed the Strengthening the Reporting of Observational Studies in Epidemiology (STROBE) guidelines for observational studies [12].

Between June 2022 and February 2024, a total of 41 living donor hepatectomies were performed at our center. One case of left hepatectomy was excluded. Of the remaining 40 right liver donors, 3 cases that underwent planned open conversion were excluded. Ultimately, 37 cases (13 in the robotic group [12 fully robotic and 1 unplanned open conversion] and 24 in the open group) were enrolled in this study, and the patient demographics, intraoperative data, and outcomes of these two groups were compared and analyzed. Following the intention-to-treat principle, we included one case of unplanned open conversion due to gas embolism in the robotic group, as this case was initially intended to undergo fully robotic approach. Data were collected from the patient’s medical and operative records.

Demographic data included age, sex, Body Mass Index (BMI), underlying liver disease, Model for End-Stage Liver Disease (MELD)-Na score of the recipients, and imaging study results. Operative data included operation time, estimated blood loss (EBL), detailed anatomy of the graft, cold ischemia time (CIT), warm ischemia time (WIT), and biliary reconstruction technique of the recipient. The first WIT was defined as the time from cross-clamping to the backtable perfusion of the graft. The second WIT was defined as the time from the removal of the liver graft from ice to reperfusion. The anatomy of the liver graft was classified using the Huang classification [13] for the bile duct and Nakamura’s classification [14] for the portal vein. Postoperative outcomes focused on donor/recipient laboratory data, complications, and hospital stay. Postoperative complications were classified using the Clavien–Dindo (CD) classification [15]. In addition, the Comprehensive Complication Index (CCI) [16] of each donor was calculated at the time of discharge and on postoperative day (POD) 30. Bile leak was evaluated according to the International Study Group for Liver Surgery (ISGLS) definitions [17]. Early allograft dysfunction (EAD) was defined according to the criteria proposed by Olthoff et al. [18] During the study period, our center underwent several changes to the perioperative analgesia protocol (including peripheral nerve block, epidural anesthesia, and intravenous patient-controlled analgesia only). Therefore, this study was unable to evaluate pain outcomes.

### Statistical analysis

Data are presented as the median (range) or number (percentage). The data between the two groups were compared using the Chi-square test or Fisher exact test for categorical variables and the Mann–Whitney *U* test for continuous variables. *P* values of < 0.05 was considered statistically significant. All statistical analyses were performed with R (version 3.6.1; The R Foundation for Statistical Computing, Vienna, Austria).

## Results

### Donor and recipient demographics

In terms of the donor demographics, there were no statistically significant differences in age, sex, total liver volume, and right liver volume between the two groups (Table 1). The BMI of the robotic group was significantly lower than that of the open group (median [range], 24.6 [20.3–35.0] vs 29.8 [20.5–34.0]; *P* = 0.002), and the fat fraction of the liver of the robotic group was significantly lower than that of the open group (median [range], 1.4 [1.0–3.8] % vs 3.4 [0.8–9.1] %; *P* = 0.002). The recipient demographics did not reveal any differences in age, sex, BMI, or MELD-Na score.

**Table 1:** Donor and recipient demographics

Variables	Robotic (*n* = 13)	Open (*n* = 24)	*P* value
Donor			
Age, median (range), years	39 (25–51)	46.5 (26–59)	0.12
Sex, No. (%)			0.29
Male	3 (23.1)	11 (45.8)	
Female	10 (76.9)	13 (54.2)	
BMI, median (range),	24.6 (20.3–35.0)	29.8 (20.5–34.0)	0.002
Previous upper abdominal surgery, No. (%)	4 (30.8)	3 (12.5)	0.21
Total liver volume, median (range), mL	1410 (1029–2092)	1530 (1173–2097)	0.08
Right liver volume, median (range), mL	931 (618–1340)	973 (778–1354)	0.30
Fat fraction on MRI, median (range), %	1.4 (1.0–3.8)	3.4 (0.8–9.1)	0.002
Recipient			
Age, median (range), years	65 (25–77)	61 (25–76)	0.73
Sex, No. (%)			0.17
Male	5 (38.5)	16 (66.7)	
Female	8 (61.5)	8 (33.3)	
BMI, median (range)	29.2 (21.2–40.1)	27.4 (20.4–42.1)	0.20
MELD-Na score, median (range)	15.0 (7–25)	16.0 (9–27)	0.17
Indication, No. (%)			
NASH	3 (23.1)	9 (37.5)	
EtOH	3 (23.1)	5 (20.8)	
HCV	3 (23.1)	2 (8.3)	
AIH	1 (7.7)	2 (8.3)	
PBC/PSC	1 (7.7)	4 (16.7)	
Retransplantation	1 (7.7)	0 (0)	
Others	1 (7.7)	2 (8.3)	

*AIH* autoimmune hepatitis, *BMI* body mass index, *EtOH* alcohol-related liver disease, *HCV* hepatitis C virus, *MELD* Model for End-Stage Liver Disease, *MRI* magnetic resonance imaging, *NASH* non-alcoholic steatohepatitis, *PBC* primary biliary cholangitis, *PSC* primary sclerosing cholangitis

### Intraoperative variables

**Fig. 2:**
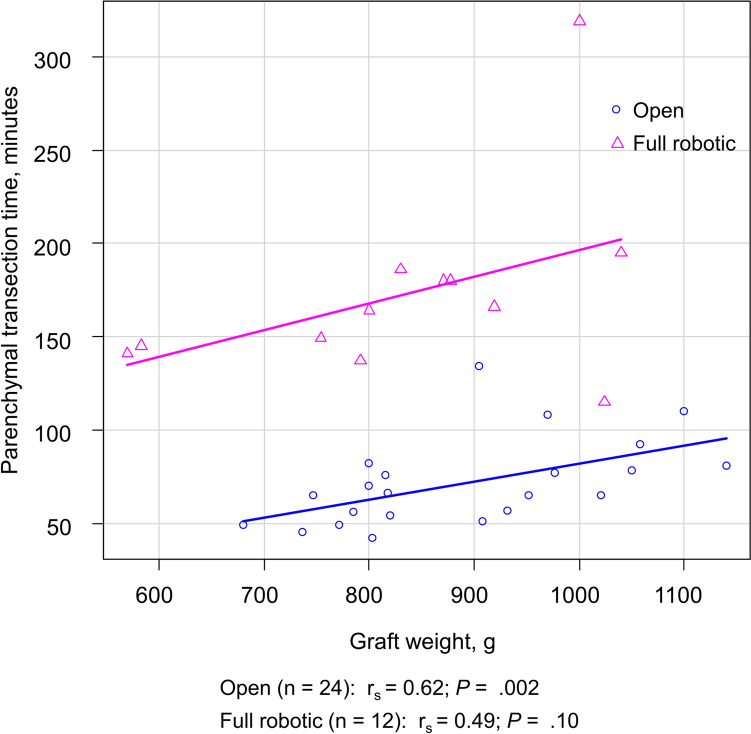
Correlation between graft weight and parenchymal transection time. A positive correlation was observed between graft weight and parenchymal transection time, with a statistically significant difference in the open group (Spearman’s rank correlation coefficient [*r*_s_] = 0.62; *P* = 0.002), whereas the fully robotic group showed a trend toward a positive correlation but without statistical significance (*r*_s_ = 0.49; *P* = 0.10)

The robotic group showed longer hilar dissection and liver mobilization time (median [range], 124 (103–151) minutes vs 96 (66–120) minutes; *P* < 0.001), parenchymal transection time (median [range], 166 [115–319] minutes vs 66 [42–134] minutes; *P* < 0.001), total operation time (median [range], 451 [374–568] minutes versus 368 [276–421] minutes; *P* < 0.001), first WIT (median[range], 8 [2–16] minutes vs 3 [1–14] minutes; *P* = 0.004), and CIT (median [range], 105 [43–164] minutes vs 73 [38–143] minutes; *P* = 0.02) compared to the open group (Table 2). A positive correlation was observed between graft weight and parenchymal transection time (Fig. 2). Blood loss was significantly lower in the robotic group compared to the open group (median [range], 200 [50–700] mL vs 900 [300–2500] mL; *P* < 0.001). Salvaged blood was returned in 54.2% (13/24) of the cases in the open group. One donor in the robotic group who required unplanned open conversion received homologous blood transfusion (310 mL of packed red blood cells) due to blood loss of 700 mL after open conversion. No other donors in either group required homologous blood transfusion. There were no significant differences in graft weight, GRWR, graft anatomy, or recipient biliary reconstruction technique between the two groups. The details of the graft anatomy are summarized in Table 2.

**Table 2:** Donor and recipient intraoperative variables

Variables	Robotic (*n* = 13)	Open (*n* = 24)	*P* value
Donor/graft			
Blood loss, median (range), mL	200 (50–700)	900 (300–2500)	< .001
Intraoperative cell salvage use, No. (%)	0 (0)	13 (54.2)	0.002
Operation time, median (range), minutes	451 (374–568)	368 (276–421)	< .001
Hilar dissection and liver mobilization time, median (range), minutes	124 (103–151)	96 (66–120)	< .001
Parenchymal transection time, median (range), minutes	166 (115–319)	66 (42–134)	< .001
First WIT, median (range), minutes	8 (2–16)	3 (1–14)	0.004
Graft with middle hepatic vein, No. (%)	0 (0)	5 (20.8)	0.14
Graft weight, median (range), g	871 (570–1040)	839 (680–1140)	0.69
GRWR, median (range), %	1.02 (0.63–1.66)	1.03 (0.66–1.65)	0.95
Number of hepatic arteries, No. (%)			0.35
1	12 (92.3)	24 (100)	
2	1 (7.7)	0 (0)	
Bile duct anatomy (Huang classification), No. (%)			0.73
A1	6 (46.2)	13 (54.2)	
A2	2 (15.4)	2 (8.3)	
A3	2 (15.4)	5 (20.8)	
A4	2 (15.4)	4 (16.7)	
Not classifiable	1 (7.7)	0 (0)	
PV anatomy (Nakamura’s classification), No. (%)			0.36
A	12 (92.3)	19 (79.2)	
B	0 (0)	4 (16.7)	
C	1 (7.7)	1 (4.2)	
Extension graft of PV, No. (%)	6 (46.2)	5 (20.8)	0.14
IRHV, No. (%)	3 (23.1)	6 (25.0)	1
Reconstruction of V5/V8, No. (%)	10 (76.9)	14 (58.3)	0.31
Recipient			
Blood loss, median (range), mL	2450 (200–11,775)	2000 (500–10,000)	0.75
Operation time, median (range), minutes	595 (438–802)	480 (416–690)	0.004
CIT, median (range), minutes	105 (43–164)	73 (38–143)	0.02
Second WIT, median (range), minutes	37 (23–48)	36 (14–60)	0.66
Bile duct reconstruction, No. (%)			0.81
Duct to duct	11 (84.6)	21 (87.5)	
Hepaticojejunostomy	1 (7.7)	2 (8.3)	
Duct-to-duct and Hepaticojejunostomy	1 (7.7)	1 (4.2)	

*CIT* cold ischemia time, *GRWR* graft-to-recipient weight ratio, *IRHV* inferior right hepatic vein, *PV* portal vein, *V5/V8* segment 5/8 veins, *WIT* warm ischemia time

Regarding recipient, operation time was significantly longer in the robotic group compared to the open group (median [range], 595 [438–802] vs 480 [416–690] minutes; *P* = 0.004). Blood loss was comparable between the two groups (median [range], 2450 [200–11775] vs 2000 [500–10000] mL; *P* = 0.75).

### Postoperative outcome

Regarding outcomes of the donors, there were no significant differences in laboratory data and hospital stays between the two groups (Table 3; detailed laboratory data are listed in Supplementary Table 2). Regarding postoperative complications, there were a total of 3 major complications (CD grade IIIB) necessitating surgical intervention: two cases of hematomas/bleeding in the robotic group and one case of incisional hernia in the open group. Regarding hematoma/bleeding in the robotic group, the first patient developed rectus sheath hematoma at the Pfannenstiel incision site and required evacuation of hematoma 2 weeks after the original surgery. This patient returned to stable afterward. The second patient developed postoperative bleeding immediately after surgery, necessitating an emergency exploratory laparotomy. One bleeding point was identified on the liver transection surface and controlled with sutures. This patient returned to stable afterward and was discharged on POD 7. All other complications were minor (CD grade I or II). There were no donor bile leaks in the robotic group and two in the open group (both were CD grade I and ISGLS grade A). There were no surgical site infections. Ileus was the most common complication in both groups, but most cases were CD grade I and resolved within a few days without any intervention. There were no differences in CCI at discharge (median [range], 8.7 [0–34.8] vs 0 [0–29.6]; *P* = 0.53) and CCI at 30 days (median [range], 8.7 [0–34.8] vs 0 [0–29.6];* P* = 0.23) between the two groups. There was no donor mortality.

**Table 3:** Postoperative outcomes

Variables	Robotic (*n* = 13)	Open (*n* = 24)	*P* value
Donor			
Peak AST, median (range), U/L	245 (147–554)	311 (114–983)	0.14
Peak ALT, median (range), U/L	269 (158–652)	333 (84–839)	0.15
Peak total bilirubin, median (range), mg/dL	3.5 (2.4–5.1)	3.2 (1.8–6.5)	0.46
Peak INR, median (range)	1.7 (1.4–1.9)	1.7 (1.2–2.0)	0.55
AST on POD 5, median (range), U/L	64 (54–132)	74 (34–236)	0.87
ALT on POD 5, median (range), U/L	124 (65–212)	125 (54–347)	0.96
Total bilirubin on POD 5, median (range), mg/dL	1.5 (0.9–1.8)	1.2 (0.5–2.8)	0.17
INR on POD 5, median (range)	1.2 (1.0–1.3)	1.1 (1.0–1.2)	0.08
Bile leak, No. (%)	0 (0)	2 (8.3) CD I/ISGLS A: *n* = 2	0.54
Bleeding/Hematoma, No. (%)	2 (15.4) *CD IIIB: *n* = 2	0 (0)	0.10
Ileus, No. (%)	7 (53.8) *CD I: * n* = 6; CD II: * n* = 1	9 (37.5) *CD I: * n* = 6; CD II: * n* = 3	0.49
Incisional hernia, No. (%)	0 (0)	1 (4.2) *CD IIIB	1
CCI at discharge, median (range)	8.7 (0–34.8)	0 (0–29.6)	0.53
CCI at 30 days, median (range)	8.7 (0–34.8)	0 (0–29.6)	0.23
Hospital stays, median (range), days	8 (5–9)	7 (5–14)	0.58
Follow-up, median (range), days	59 (13–189)	201 (28–464)	
Recipient			
Peak AST, median (range), U/L	383 (123–725)	286 (120–1630)	0.35
Peak ALT, median (range), U/L	336 (194–888)	311 (99–1010)	0.36
Total bilirubin on POD 7, median (range), mg/dL	1.8 (0.3–10.1)	1.5 (0.5–18.5)	0.81
INR on POD 7, median (range)	1.1 (1.0–1.6)	1.2 (1.0–1.9)	0.29
EAD, No. (%)	1 (7.7)	3 (12.5)	1
Bile leak, No. (%)	3 (23.0)	3 (12.5)	0.38
Biliary stricture, No. (%)	1 (7.7)	3 (12.5)	1
Bleeding, No. (%)	1 (7.7)	4 (16.7)	0.64
Follow-up, median (range), days	122 (13–381)	345 (14–630)	

*ALT* alanine aminotransferase, *AST* aspartate aminotransferase, *CD* Clavien–Dindo classification, *CCI* Comprehensive Complication Index, *EAD* early allograft dysfunction, *INR* international normalized ratio, *POD* postoperative day

In terms of recipient outcomes, there were no significant differences in laboratory data, EAD rate, or complication profile between the two groups. There was no hepatic artery thrombosis, primary non-function, or 90-day graft failure/mortality in either group. There were two recipient mortalities in the open group during the follow-up period. One recipient died at 5-month post-transplantation due to persistent COVID-19 infection complicated by multiple infections, including candidemia and enterococcal bacteremia, which led to acute kidney injury and malnutrition. The second recipient died at 7-month post-transplantation due to recurrent bacterial pneumonia complicated by acute kidney injury, deconditioning, and malnutrition. No mortality was observed in the robotic group during the follow-up period.

## Discussion

Our study revealed that although the robotic approach resulted in longer operation times, it was associated with less blood loss, aligning with previous reports [6, 10, 19]. Additionally, although the first WIT and CIT were longer in the robotic group, these factors did not adversely affect the postoperative liver function of either donors or recipients. The short-term outcomes and complication rates for both donors and recipients were comparable between the two groups, and the median CCI of our cohort was below the benchmark value of 27.9 proposed by Rössler et al. [20] Therefore, we conclude that robotic living donor right hepatectomy is safe and feasible. In the following sections, we will discuss the benefits and risks of the robotic approach compared to the open approach.

One of the advantages of the robotic approach, as revealed in this study, is the reduction in blood loss. We use either the crush clamp technique or cavitron ultrasonic surgical aspiration (CUSA, Integra) in open donor hepatectomy. However, due to the unavailability of CUSA on the robotic platform, we primarily utilized the Harmonic scalpels and the Micro bipolar forceps in the robotic approach. Although the robotic Harmonic scalpel lacks an articulating function and coordinating two instruments simultaneously may have a steep learning curve, this technique is highly effective once mastered. The reduced blood loss can also be attributed to the magnified visualization, which allows for precise hemostasis and the effect of pneumoperitoneum.

The second advantage is that the robotic approach offers benefits in handling complex biliary anatomies. Bile leak and biliary strictures are among the most common complications after open or laparoscopic living donor hepatectomy [20–22]. Conversely, although there was no statistically significant difference, the incidence of biliary complications in our robotic group was zero despite more than half of the donors having multiple bile duct openings (Huang A2, A3, or A4). This success is attributed to the magnified view and ICG cholangiography, which aid in precise bile duct identification and careful dissection, thus reducing the risk of bile duct devascularization, as highlighted by Schulze et al. [10] Furthermore, our experience indicates that the robotic approach is capable of managing various vascular anatomic variations, including Nakamura type C portal vein, multiple arteries, and the presence of an inferior right hepatic vein. A 30-degree magnified camera inserted from the caudal side allows for observation of the liver hilum from various angles, enabling a more meticulous hilar dissection. Given these findings, we currently do not impose any restrictions regarding biliary and vascular variation when considering a robotic approach.

Regarding the risks of the robotic approach, we must first discuss the early case that required open conversion due to concerns of gas embolism. In this case, toward the end of the parenchymal transection, the patient’s blood pressure suddenly dropped without any evidence of bleeding, and the end-tidal CO2 and SpO2 also dropped. The patient was quickly stabilized with the cessation of pneumoperitoneum and hemodynamic support by the anesthesia team. However, as this was among our initial cases, we decided to convert to open surgery for safety. Although severe cases are rare, previous reports [23] indicate that the incidence of gas embolism is higher in laparoscopic hepatectomy compared to other laparoscopic surgeries (1.2%–4.5%). It is crucial to recognize the risk of gas embolism and to be prepared with an appropriate response. After experiencing this case, we set the upper limit of pneumoperitoneum pressure to 12 mmHg, and we have not encountered any clinically significant cases of gas embolism since then.

Second, there were two cases of bleeding complications (hematoma at the Pfannenstiel incision site and bleeding from the transection surface) requiring surgical intervention in the robotic group. Wound hematoma at the Pfannenstiel incision has been reported as a common complication (2.4%) in robotic donor hepatectomy [10]. This experience prompted us to pay closer attention to hemostasis during the closure of the Pfannenstiel incision. In the second case, it is possible that the pneumoperitoneum pressure masked the bleeding. Since experiencing this case, we have made it a practice to lower the pneumoperitoneum pressure and perform a final check for hemostasis before closure.

Our experience is unique in that it highlights the feasibility of adopting robotic donor hepatectomy programs, even in U.S. centers like ours, where the volume of LDLT and HPB cases is not as high, and with no prior experience with the laparoscopic approach as a team. However, it is important to emphasize that successful implementation of a robotic living donor right hepatectomy program requires extensive preparation and experience. In our center, the console surgeon had substantial experience in both open donor hepatectomy and robotic liver resection before initiating the robotic donor program. The surgical team spent one year preparing for the robotic donor program with a fixed team. Additionally, the team visited high-volume centers to observe their robotic donor programs. We implemented a stepwise approach to program initiation, beginning with planned hybrid procedures (planned open conversion) for the initial cases before transitioning to complete robotic procedures. This gradual implementation strategy, combined with the surgeon’s prior experience and dedicated team preparation, was crucial for ensuring donor safety during introducing this new technique.

Our study has several limitations. First, although it is the largest series reported from the USA, it is a retrospective study that includes a relatively small sample size. Second, our focus was on short-term outcomes, necessitating further research into long-term outcomes for both donors and recipients. Third, we did not investigate aspects of donor QOL, such as postoperative pain and patient satisfaction in terms of cosmetic outcomes. A large-scale prospective study that includes research on donor QOL is necessary. Fourth, while left liver grafts are associated with fewer donor complications than right liver grafts, due to relatively high BMI of recipients and limited experience with left liver grafts, right liver was primarily utilized in our center. Further studies are needed to evaluate the safety and feasibility of robotic living donor left hepatectomy.

In conclusion, compared to the open approach, robotic living donor right hepatectomy involved longer operative times but resulted in less blood loss. The short-term outcomes and complication rates were comparable. Our experience demonstrates that both the surgeon’s extensive experience in open donor hepatectomy and robotic surgery, as well as the meticulous preparation as a team, are essential for the safe implementation of a robotic donor hepatectomy program.

## Supplementary Information

Below is the link to the electronic supplementary material.

Supplementary file1 (DOCX 22 kb)

Supplementary file2 (DOCX 24 kb)

## Data Availability

The data that support the findings of this study are available from the corresponding author upon reasonable request.

## Abbreviations

BMI: Body mass index
CCI: Comprehensive Complication Index
CD: Clavien–Dindo
CIT: Cold ischemia time
CT: Computed tomography
CUSA: Cavitron ultrasonic surgical aspiration
EAD: Early allograft dysfunction
EBL: Estimated blood loss
GRWR: Graft-to-recipient weight ratio
HPB: Hepato-pancreato-biliary
ICG: Indocyanine green
ISGLS: International Study Group for Liver Surgery
LDLT: Living donor liver transplantation
MELD: Model for End-Stage Liver Disease
MRCP: Magnetic resonance cholangiopancreatography
MRI: Magnetic resonance imaging
POD: Postoperative day
QOL: Quality of life
STROBE: Strengthening the Reporting of Observational Studies in Epidemiology
WIT: Warm ischemia time
