# Supplementary material for: Early outcomes of robotic vs open living donor right hepatectomy in a US Center
Source: Surg Endosc. 2025 Jan 8;39(3):1643–52. doi: 10.1007/s00464-024-11469-4 (PMC11870880; doi:10.1007/s00464-024-11469-4)
Supplement: Supplementary file 1 — Supplementary file1 (DOCX 22 kb) [file 464_2024_11469_MOESM1_ESM.docx]

Supplementary Table 1. Selection criteria for robotic living donor right hepatectomy at Virginia Commonwealth University Hume-Lee Transplant Center

|  | Initial criteria (Until 4th case) | Current criteria |
| --- | --- | --- |
|  |  |  |
| Age | < 40 years | < 60 years |
| BMI | < 25 | < 35 |
| Previous abdominal surgery | No upper abdominal surgery | — |
| Estimated graft volume | < 800 mL | < 1200 mL |
| Hepatic artery | Single | — |
| Portal vein | Single (Nakamura A), length > 1 cm | — |
| IRHV | No significant (> 5 mm) IRHV | — |
| Bile duct | Single (Huang A1) | — |

Abbreviations: BMI, body mass index; IRHV, inferior right hepatic vein.
