# Supplementary material for: Early outcomes of robotic vs open living donor right hepatectomy in a US Center
Source: Surg Endosc. 2025 Jan 8;39(3):1643–52. doi: 10.1007/s00464-024-11469-4 (PMC11870880; doi:10.1007/s00464-024-11469-4)
Supplement: Supplementary file 2 — Supplementary file2 (DOCX 24 kb) [file 464_2024_11469_MOESM2_ESM.docx]

Supplementary Table 2. Laboratory data

| Variables | Robotic  (n = 13) | Open (n = 24) | *P* value |
| --- | --- | --- | --- |
|  |  |  |  |
| Donor |  |  |  |
| Peak AST, median (range), U/L | 245 (147—554) | 311 (114—983) | .14 |
| Peak ALT, median (range), U/L | 269 (158—652) | 333 (84—839) | .15 |
| Peak Total bilirubin, median (range), mg/dL | 3.5 (2.4—5.1) | 3.2 (1.8—6.5) | .46 |
| Peak INR, median (range) | 1.7 (1.4—1.9) | 1.7 (1.2—2.0) | .55 |
| AST on POD 3, median (range), U/L | 85 (44—148) | 80 (41—175) | .87 |
| ALT on POD 3, median (range), U/L | 137 (68—278) | 150 (44—393) | .81 |
| Total bilirubin on POD 3, median (range), mg/dL | 2.0 (1.1—3.1) | 2.0 (0.7—4.0) | .59 |
| INR on POD 3, median (range) | 1.3 (1.1—1.5) | 1.2 (1.1—1.4) | .08 |
| AST on POD 5, median (range), U/L | 64 (54—132) | 74 (34—236) | .87 |
| ALT on POD 5, median (range), U/L | 124 (65—212) | 125 (54—347) | .96 |
| Total bilirubin on POD 5, median (range), mg/dL | 1.5 (0.9—1.8) | 1.2 (0.5—2.8) | .17 |
| INR on POD 5, median (range) | 1.2 (1.0—1.3) | 1.1 (1.0—1.2) | .08 |
| Recipient |  |  |  |
| Peak AST, median (range), U/L | 383 (123—725) | 286 (120—1630) | .35 |
| Peak ALT, median (range), U/L | 336 (194—888) | 311 (99—1010) | .36 |
| Peak INR, median (range) | 2.1 (1.5—2.9) | 2.05 (1.5—4.3) | .74 |
| Total bilirubin on POD 7, median (range), mg/dL | 1.8 (0.3—10.1) | 1.5 (0.5—18.5) | .81 |
| INR on POD 7, median (range) | 1.1 (1.0—1.6) | 1.2 (1.0—1.9) | .29 |

Abbreviations: ALT, alanine aminotransferase; AST, aspartate aminotransferase; INR, international normalized ratio; POD, postoperative day.
